# Supplementary material for: Dietary Polyacetylenic Oxylipins Falcarinol and Falcarindiol Prevent Inflammation and Colorectal Neoplastic Transformation: A Mechanistic and Dose-Response Study in A Rat Model
Source: Nutrients. 2019 Sep 14;11(9):2223. doi: 10.3390/nu11092223 (PMC6769548; doi:10.3390/nu11092223)
Supplement: Supplementary file 1 [file nutrients-11-02223-s001.pdf]

Supplementary Materials

Dietary polyacetylenic oxylipins falcarinol and falcarindiol prevent inflammation and colorectal neoplastic transformation through COX-2 inhibition: A mechanistic and dose-response study in a rat model

Morten Kobaek-Larsen [1,2,*], Gunnar Baatrup [1,2], Martine K. Notabi [3], Rime B. El-Houri [3], Emma Pipó-Ollé [3], Eva C. Arnspang [3] and Lars P. Christensen [4]

1 Department of Clinical Research, University of Southern Denmark, 5000 Odense, Denmark
2 Department of Surgery, Odense University Hospital, 5000 Odense, Denmark
3 Department of Chemical Engineering, Biotechnology and Environmental Technology, University of Southern Denmark, 5230 Odense M, Denmark
4 Department of Chemistry and Bioscience, Faculty of Engineering and Science, Aalborg University, 6700 Esbjerg, Denmark

* Correspondence: Morten.Kobaek.Larsen@rsyd.dk; Tel.: Tel: +45 2461 3161

Table S1. Primer sequences used for analysis of gene expression.

Gene	Official gene name	Forward Primer (5'-3')	Revers Primer (5'-3')	Probe (5'-3')
COX-1	PTGS1	GTAGACCTTGGCCACATTTATG	AAGTTTCCCATCCTTGAAGAGC	TCTGGAZACGACAZGTACCZACC
COX-2	PTGS2	CCACCCCAAACACAGTACAC	CGAAGGAAGGGAATGTTGTTCA	CCACTTCZAAGGGAGTCTGZGA
NF-κβ	NFKB1	ACACTTAGCCATCATCCACCT	TGTCATCAGAGATCGAACCAG	GCTCGTZGAGGGATCTZGCTAG
IL-1β	IL1B	TCCTGTGTGATGAAAGACGG	GGGAACTGTGCAGACTCAAA	CTGGAGZAGTGTGGZATCCCAA
IL-6	IL6	CCACCCACAACAGACCAGTA	TCTCATTTCCAAGATCTCCCTGA	ACCACTTCACZAAGTCGGAZGG
PPARγ2	PPARG	GGAATCAGCTCTGTGGACCT	GCTCTTGTGAACGGGATGTC	TGGATGZACCZACTCCCZATTCC
TNFα	TNF	CACCACGCTCTTCTGTCTAC	GGGCTTGTCACTCGAGTTTT	GGTCCCAACAAGZGAGGAGZAA

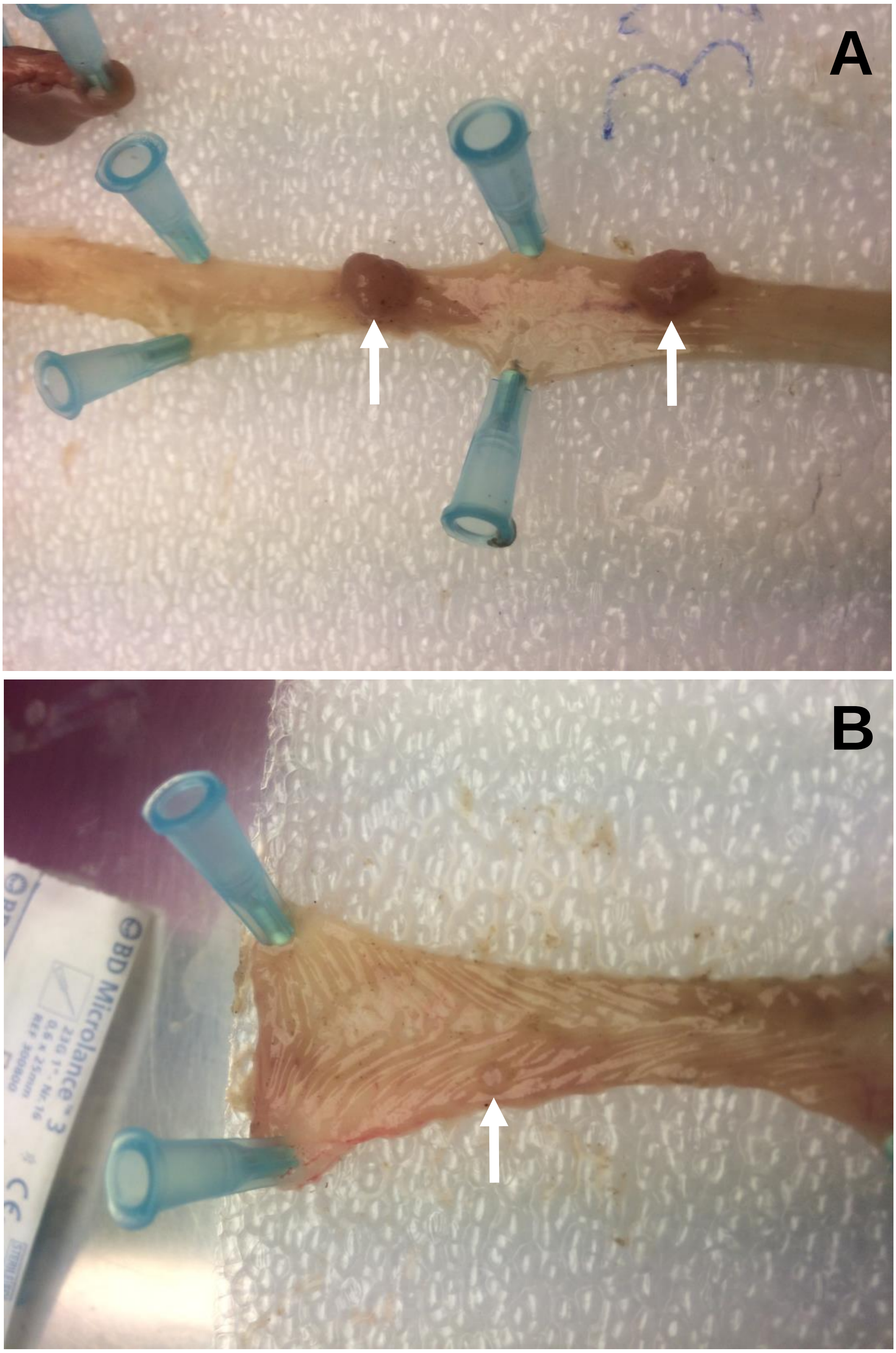

Figure S1. The macroscopic polyp lesions in the AOM-treated rats varied in size from (**A**) large adenomas to (**B**) small adenomas. The adenomas was in general smaller in rats on a SRD supplemented with FaOH and FaDOH compared to rats on a SRD (control group). In addition, the percentage of large adenomas of the total macroscopic polyp lesions was highest in the control group.
